# Phlebitis Cured by the Constant Application of Snow

**Published:** 1846-04

**Authors:** 


*Phlebitis cured by the constant application of Snow.*—A man, aged sixty, of robust constitution, was bled in the right arm, November 30th, 1840, for the removal of plethora. Next day he felt a tightness in the bend of the limb ; the small wound became tense and inflamed, and began to suppurate. On the 10th of December intense fever, succeeded by delirium, declared itself. Dr. Margariteau was called in on the 17th. The disease was then making progress ; the arm and fore-arm considerably swollen, and of an erysipelatous hue ; and the vein, in its course to the axilla, indurated, and of the volume of a finger. Forty leeches, followed by emollient cataplasms, were applied to the fore-arm ; an opiate potion and rigorous abstinence prescribed. 18th, March the same, no delirium during the night ; fever as violent as ever. Night of the 19th ; much worse ; the delirium returned with aggravated violence. Forty leeches were again applied to the limb, and the opiate potion continued. The inflammation, notwithstanding, went on to such an extent that the integument of the whole arm assumed a livid colour, and began to separate ; and all the formidable signs of a speedily fatal termination were developed. At this time it was determined to combat the inflammatory action by enveloping the whole limb in cataplasms of snow, which then covered the earth ; care being taken to renew them night and day as the snow liquefied. This treatment was commenced on the 20th of December; its effects were marvellous. During the first twenty-four hours, the pains of the limb disappeared ; the fever and delirium abated ; and the patient passed a tranquil night. Next day, the tension and redness were considerably less ; and all the constitutional symptoms alleviated. After six days' unremitting use, the patient could no longer bear the application of the snow, and it was discontinued. The danger was then past. There was no fever ; and, although the arm remained swollen, the sense of heat and pain had disappeared. From this moment, recourse was had to methodical compression by means of a spiral bandage applied from the fingers to the axilla, and moistened with Goulard-water (Aqua de vegeto.)

After this, the tumefaction of the arm gradually subsided; but a degree of induration remained in the whole track of the vein, more especially in the axillary region. And, although, for the space of six months, the limb could not be moved without pain, the patient regained his health, with no vestige of the injury remaining, except some enlarged superficial veins.—*The Medical Times.*

